# Global Socioeconomic Impact of Cystic Echinococcosis

**DOI:** 10.3201/eid1202.050499

**Published:** 2006-02

**Authors:** Christine M. Budke, Peter Deplazes, Paul R. Torgerson

**Affiliations:** * University of Zürich, Zürich, Switzerland

**Keywords:** echinococcosis, cestodes, cost of illness, burden of illness, economics, zoonoses

## Abstract

Cystic echinococcosis (CE) is an emerging zoonotic parasitic disease throughout the world. Human incidence and livestock prevalence data of CE were gathered from published literature and the Office International des Epizooties databases. Disability-adjusted life years (DALYs) and monetary losses, resulting from human and livestock CE, were calculated from recorded human and livestock cases. Alternative values, assuming substantial underreporting, are also reported. When no underreporting is assumed, the estimated human burden of disease is 285,407 (95% confidence interval [CI], 218,515–366,133) DALYs or an annual loss of US $193,529,740 (95% CI, $171,567,331–$217,773,513). When underreporting is accounted for, this amount rises to 1,009,662 (95% CI, 862,119–1,175,654) DALYs or US $763,980,979 (95% CI, $676,048,731–$857,982,275). An annual livestock production loss of at least US $141,605,195 (95% CI, $101,011,553–$183,422,465) and possibly up to US $2,190,132,464 (95% CI, $1,572,373,055–$2,951,409,989) is also estimated. This initial valuation demonstrates the necessity for increased monitoring and global control of CE.

## Abstract 2

Because the human and economic losses of cystic echinococcosis are substantial, global prevention and control measures should be increased.

Cystic echinococcosis (CE) is a condition of livestock and humans that arises from eating infective eggs of the cestode *Echinococcus granulosus*. Dogs are the primary definitive hosts for this parasite, with livestock acting as intermediate hosts and humans as aberrant intermediate hosts. The outcome of infection in livestock and humans is cyst development in the liver, lungs, or other organ system. The distribution of *E*. *granulosus* is considered worldwide, with only a few areas such as Iceland, Ireland, and Greenland believed to be free of autochthonous human CE. However, CE is not evenly distributed geographically (Figure 1) (*1*). For example, the United States has few cases in livestock and most human cases are imported. The same is true for regions of Western and Central Europe. In many parts of the world, however, CE is considered an emerging disease. For example, in the former Soviet Union and Eastern Europe, the number of observed cases has dramatically increased in recent years (*2**–**4*). Additionally, in other regions of the world, such as parts of China, the geographic distribution and extent of CE are greater than previously believed (*5*). CE not only causes severe disease and possible death in humans, but also results in economic losses from treatment costs, lost wages, and livestock-associated production losses. To date, no global estimates exist of CE burden (total health, socioeconomic, and financial cost of a given disease to society) in humans or livestock. Such an estimate is imperative since it can be used as a tool to prioritize control measures for CE, which is essentially a preventable disease.

**Figure 1:**
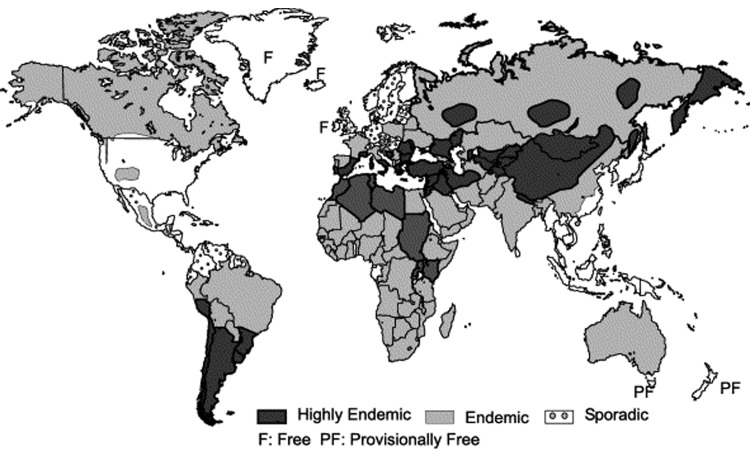
Global distribution of zoonotic strains of *Echinococcus granulosus*. (Adapted from Eckert and Deplazes, 2004 (*1*). Copyright Institute für Parasitologie, Universität Zürich; used with permission.)

Two methods previously used to assess disease burden are disability adjusted life years (DALYs) and the calculation of monetary losses (*6*). DALYs were first developed in the 1990s and were used in the Global Burden of Disease (GBD) Study to determine the worldwide burden of disease due to both communicable and noncommunicable causes (*7*). Although the application of DALYs is becoming more commonplace, the use of DALYs and the methods behind the creation of this measure remain debatable (*8*). The GBD Study was an extensive undertaking; however, echinococcosis was not among the conditions studied. Nevertheless, DALYs have been applied to cystic echinococcosis and alveolar echinococcosis, caused by *E*. *multilocularis*, on a small scale in western China (*9*). Likewise, monetary evaluations have been applied to CE infections in humans and livestock only at a local level (*10**–**14*). Global burden indicators not only give an idea of the scope of the disease under study, but can also be used to direct limited financial resources to sites where they can be most effective. Because of the magnitude of applying burden of disease measurements on a global scale, this study must be considered a preliminary estimate. Nevertheless, this report should increase awareness of the global impact of CE by both the public health and livestock sectors.

## Materials and Methods

### CE Incidence in Humans

Data on country-specific annual reported human CE cases were obtained from the Office International des Epizooties (OIE), World Health Organization Handistatus II database for the years 1996–2003 (*15*). This information was then merged with published case reports from numerous countries and logged into an Excel spreadsheet (Microsoft Corp., Redmond, WA, USA). Type and quality of incidence data varied by country or region; however, most data consisted of annual numbers of detected cases per susceptible population or was converted into this form for analysis purposes. If both an OIE-reported and a literature-based value were available, the larger of the 2 was used. However, if the higher value appeared to be from a survey that evaluated a highly disease-endemic region and was, therefore, not applicable to the entire country, a corresponding adjustment was made. In addition, we assumed that ≈10% of annual cases are not officially diagnosed, and those patients do not receive medical attention because of their socioeconomic status or the subclinical nature of the illness. Based on past studies, this estimate is most likely conservative (*12**,**14*). For example, in China, mass ultrasound screening in remote areas has shown high prevalence rates of CE (*9*). A number of these patients have advanced clinical disease but would not normally have access to treatment because of poverty and distance from medical facilities. Human cases of CE are also systematically underreported by the healthcare establishment, with up to 75% of clinic or hospital-diagnosed cases never recorded in local or national databases or published reports (*16**,**17*). Therefore, adjustments were made to account for the substantial underreporting of known treated cases.

### CE Prevalence in Livestock

Numbers of annual reported CE cases in slaughtered livestock (sheep, goats, cattle, camels, and swine) for the years 1996–2003 were obtained from the OIE-Handistatus II database (*15*). This information was merged with abattoir studies performed in numerous countries. If data from both sources were available, the larger of the 2 estimates was used. However, if the higher value appeared to be from a region that was highly disease-endemic and was not appropriate for a countrywide estimate, an adjustment was made. Prevalence per species, for each country, was applied to the estimated number of slaughtered animals per year, with 2004 livestock numbers obtained from the FAOSTAT database (*18*). The assumption was made that approximately one fourth of sheep and goat populations, one sixth of cattle and camel populations, and the entire swine population would be slaughtered annually, based on estimated average species' lifespan (e.g., approximately one fourth of a country's sheep population would be slaughtered annually, with a typical animal life expectancy of 4 years). Such a general estimate was used because of the large amount of variation in animal production practices between and within countries. As with the human incidence data, the true number that were positive for *E*. *granulosus* at slaughter is substantially higher than reported. Therefore, a correction factor was used to estimate true prevalence.

### Application of DALYs to Human Incidence Data

The DALY formula (shown below) was applied to global human incidence data.
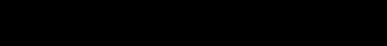

In this equation, *D* is a disability weight, *β* is an age-weighting function parameter, *C* is an age-weighting correction constant, *r* is a discount rate, *a* is age at clinical onset, and *L* is the duration of disability or time lost because of death (*7*). Disability weight for CE was assigned a multinomial distribution based on numerous retrospective studies evaluating postoperative outcome (Table 1) (*19**–**24*). The percentage of patients projected to improve after surgery was assigned a disability weight of 0.200 (Dutch weight for clinically disease-free cancer) for 1 year, the percentage of patients projected to have substantial postsurgical conditions was assigned a disability of 0.239 (GBD weight for preterminal liver cancer) for 5 years, the percentage of patients projected to have recurrent disease was assigned a disability of 0.809 (GBD weight for terminal liver cancer) for 5 years, and the percentage of patients projected to die postoperatively were assigned a disability of 1 (indicating death) for the remainder of their predicted lifespan (*7**,**25*). An assumption was also made that ≈10% of cases are not reported and do not receive medical treatment. These cases were assigned a disability weight of 0.200 (Dutch weight for clinically disease-free cancer) for 10 years (*25*). For the GBD Study, a standardized life table was used for *L* (*7*).

**Table 1:** Outcome of surgery for cystic echinococcosis in humans

Country (y)	No. patients	Cure (%)	Morbidity (%)	Relapse (%)	Death (%)	Reference
Greece (1984–1990)	56	40 (72)	13 (23)	3 (5)	0	(*9*)
Italy (1950–1987)	298	244 (82)	27 (9)	15 (5)	12 (4)	(*20*)
Turkey (1992–1999)	95	32 (34)	38 (40)	24 (25)	1 (1)	(*21*)
Turkey (1990–1995)	108	88 (81)	19 (18)	0	1 (1)	(*22*)
Greece (1985–1990)	67	59 (86)	4 (6)	3 (6)	1 (2)	(*23*)
Italy (1982–1994)	89	70 (79)	17 (19)	1 (1)	1 (1)	(*24*)
Total	713	533 (75)	118 (17)	46 (6)	16 (2)	

### Economic Evaluation of Human-associated Losses

Overall cost per human surgical case was based on findings from previous international studies (Table 2) (*11**,**13**,**14**,**26**,**27*). Expenses taken into consideration included diagnostic costs, surgical cost, hospitalization, and postoperative costs. The average cost per surgical patient was shown to be significantly correlated (R^2^ = 0.898, p < 0.05), with the country-specific per capita gross national income (per capita GNI) (Atlas Method) (Table 2). Therefore, the linear regression coefficient was used as a predictor of treatment costs for each disease-endemic country. In addition to medical costs and single-year wage losses, past studies have indicated an average 2.2% postoperative death rate for surgical patients (Table 1). Approximately 6.5% of cases also are assumed to relapse and require a prolonged recovery time (Table 1) (*11*). Therefore, these outcomes were also taken into account. We assumed that, in addition to surgical cases, ≈10% of cases are not officially diagnosed each year, and those patients never receive treatment. Wage losses for this group were thus taken into consideration. Economic losses in humans were also evaluated, taking into account the nearly 4-fold degree of underreporting of patients who received treatment.

**Table 2:** Average cost per surgical case of cystic echinococcosis

Country	Years	Average cost per case (US $)	% of real per capita GNI* per patient	Reference
Jordan	2002	701.50	40	(*26*)
Spain	1987–2001	10,915.00	76	(*27*)
Tunisia	2000	1,481.00	71	(*11*)
Uruguay	2000	6,721.00	110	(*14*)
Wales, UK	2000	13,600.30	54	(*13*)

*World Bank Atlas method for converting data in national currency to US dollars; GNI, gross national income.

### Economic Evaluation of Livestock-associated Losses

Production-based losses attributable to infected sheep, goats, cattle, camels, and pigs were estimated. Losses from liver condemnation, defined as the action of preventing the sale of livers deemed unfit for human consumption (sheep, goats, cattle, pigs, camels), reduction in carcass weight (sheep, goats, cattle), decrease in hide value (sheep, cattle), decrease in milk production (sheep, goats, cattle), and decreased fecundity (sheep, goats, cattle) were taken into account. Only liver-associated losses in camels and pigs are presented since few studies have evaluated production losses from echinococcosis in these species (*28*). Losses from liver condemnation are assumed to occur since hepatic pathology is associated with infection in swine and camels (*29*). Losses from liver condemnation were presumed proportional to those used for the analysis of the economic impact of CE in Jordan (*12*). Decrease in hide value (20%) and decrease in fecundity (11%) were presumed proportional to values suggested by numerous Soviet studies conducted from the 1950s through the 1980s (*28*). Reductions in carcass weight (2.5%) and milk production (2.5%) were also based on previous reports (*30*).

### Analysis

Spreadsheet models were constructed in Excel to estimate global impact of CE in terms of DALYs and monetary losses. Total disease effects, in DALYs lost or monetary costs, was calculated by summing all of the constituent components. Uncertainty in parameter estimates was modeled by using Monte Carlo techniques (*6*). Briefly, all parameters were assigned a probability distribution based on the quantity and quality of reported data. Macros were written in Excel to sample across these distributions, with 10,000 iterations of each model calculated. Mean and 95% confidence intervals (CIs) for losses were then determined from these iterations.

Reported global human incidence was assigned a normal distribution, with a standard deviation of 5%. Adjustments were then made to account for the nearly 4-fold degree of underreporting of treated cases believed to occur (*16**,**17*). In addition, cases that would not be officially acknowledged had to be accounted for, i.e., cases in persons who never receive treatment in a hospital. We therefore assumed that ≈10% (uniform distribution of 8% to 12%) of cases would not be detected. This estimate is conservative compared to other country-specific estimates (*12**,**14*).

The DALY formula was applied to worldwide CE cases in a stochastic manner similar to that used to apply DALYs to echinococcosis cases in a region of western China (*10*). Mean age of clinical onset (*a*) was allocated a uniform distribution of 30 to 40 years, established on the basis of various studies (Table 3) (*4**,**9**,**21**,**31**–**34*). Numerous and varying reports have indicated the sex of CE-positive persons with women tending to be infected at a higher rate than men. Based on these reports, we assigned a uniform distribution of 50% to 60% of infected persons as female (*4**,**35*). Number of DALYs lost, using incidence values corrected and uncorrected for underreporting of surgical incidence, was determined.

**Table 3:** Average age at ultrasound detection or surgery

Country	Years	Average age at onset/detection (y)	Reference
China	2001–2003	35*	(*9*)
Jordan	1994–2000	31–45†	(*31*)
Kenya (Turkana)	1979–1982	21–30*	(*32*)
Kyrgyzstan	1991–2000	22†	(*4*)
Morocco	2000–2001	32*	(*33*)
Turkey	1992–1999	44†	(*21*)
Uruguay	1991–1992	45*	(*34*)

*Age at time of ultrasound detection. †Age at surgery.

Human-associated economic losses were applied in a stochastic manner similar to that used for a region of western China (*10*). Variability in surgical treatment costs, due to CE, was modeled by using a uniform distribution of 50% to 90% of per capita GNI per country and was weighted by each country's contribution to global human CE incidence (*36*). Lower income, higher unemployment, or both has been associated with a diagnosis of CE (*4**,**10*). Consequently, a decrease in wages earned was assumed, at least for the year of initial diagnosis and treatment. Therefore, all patients were assigned a uniform loss of 50% to 90% of country-specific per capita GNI for 1 year (*36*). Approximately 6.5% of patients were also assigned a 50%–90% wage loss for 4 additional years because of relapse and prolonged recovery time. In addition, 2.2% of patients were assigned a 100% wage loss until the expected retirement age of 65 due to postsurgical death. A standard 3% discounting rate was applied to all income losses (*7*). In addition to surgical cases, ≈10% of cases (uniform distribution of 8% to 12%) annually were assumed to not be officially diagnosed. A 25% wage loss for 5 years was consequently assigned to this population. This estimate is conservative and does not take into account income losses attributable to undiagnosed cases with fatal outcomes. Projections were made that assumed the absence and presence of underreporting of surgical incidence (*16**,**17*). In addition to using real per capita GNI (Atlas Method), calculations were also performed by using purchasing power parity (ppp) adjusted per capita GNI.

As with human-associated economic losses, livestock-associated losses were applied in a stochastic manner (*10*). Livestock prices were given uniform distributions of US $30–$60 for sheep, US $15–$30 for goats, US $150–$350 for cattle, US $300–$600 for camels, and US $55–$75 for pigs. Uniform distributions were used because of the large regional variations in prices and assigned in accordance with baseline prices for most affected countries. Production losses were assumed to follow a log-normal distribution; most affected animals were lightly infected, and only a small proportion of animals had severe losses. As with human cases, substantial underreporting of livestock infection was recognized, since official reporting is not mandatory in most countries. Therefore, a uniform correction factor of 1.5 to 2 was used to approximate true economic losses. A large uniform distribution was used because of the lack of information concerning true global prevalence of CE in livestock. This lack will, therefore, be represented in the wide confidence limits obtained.

## Results

### DALYs

Regional findings for predicted global burden of CE in terms of DALYs lost, with 95% CIs, can be found in Table 4. The most conservative estimate of number of global DALYs lost is 285,407 (95% CI, 218,515–366,133), with no consideration for disease underreporting. Estimated number of global DALYs lost, taking into consideration nonreported surgical cases, is 1,009,661 (95% CI, 862,119–1,175,654).

**Table 4:** Estimated global impact of cystic echinococcosis in terms of DALYs lost

Region*	Total unadjusted DALYs lost (95% CI)†	Total adjusted DALYs lost (95% CI)
Western Europe, USA, Canada, Australia, New Zealand	11,842 (8,977–15,722)	41,891 (30,949–55,014)
Middle Eastern Crescent	104,503 (79,291–135,722)	370,056 (275,228–486,353)
Formerly socialist economies of Europe and Russia	17,317 (13,129–22,371)	61,369 (45,800–80,077)
China	112,451 (85,001–145,898)	398,015 (295,922–521,879)
Other Asia and Islands	1,130 (851–1,462)	4,003 (2,971–5,256)
Sub-Saharan Africa	2,639 (1,926–3,518)	9,314 (6,664–12,623)
Latin America and the Caribbean	14,834 (11,252–19,241)	52,693 (38,787–69,380)
India	20,691 (15,666–26,822)	73,364 (54,518–96,263)
World	285,407 (218,515–366, 133)	1,009,662 (862,119–1,175,654)

*Regional breakdown of disability-associated life years (DALYs) lost is based on that used in the Global Burden of Disease study (*7*). †CI, confidence interval.

### Human-associated Economic Losses

Findings for predicted regional burden of human CE in economic terms, with 95% CI, can be found in Table 5. Global losses, assuming no underreporting, are estimated at US $193,529,740 (95% CI, $171,567,331–$217,773,513). Losses, adjusted for underreporting, are estimated at US $763,980,979 (95% CI, $676,048,731–$857,982,275). When ppp adjusted per capita GNI is used instead of real per capita GNI, estimated annual overall losses, without correction for underreporting, are US $484,878,359 (95% CI, $432,898,134–US $542,048,125). When corrected for underreporting, annual losses are estimated at US $1,918,318,955 (95% CI, $1,700,574,632–$2,142,268,992) (Table 5).

**Table 5:** Global annual cystic echinococcosis–associated economic losses to humans

Region	Total adjusted economic losses (95% CI)*(US $)	Total adjusted economic losses (95% CI)† (US $)
Western Europe, USA, Canada, Australia, New Zealand	$309,983,585 ($244,256,327–$383,371,741)	$354,460,281 ($277,178,852–$440,438,597)
Middle Eastern Crescent	$197,276,106 ($158,870,204–$240,282,181)	$564,496,304 ($454,402,304–$690,682,060)
Formerly socialist economies of Europe and Russia	$46,896,902 ($37,750,210–$57,355,873)	$143,921,865 ($114,323,294–$176,555,114)
China	$146,129,578 ($114,279,187–$181,937,463)	$663,712,150 ($516,048,103–$826,353,341)
Other Asia and Islands	$1,535,990 ($1,159,946–$1,946,632)	$2,412,386 ($1,826,342–$3,074,240)
Sub-Saharan Africa	$832,295 ($649,915–$1,035,681)	$5,176,229 ($3,710,869–$6,969,680)
Latin America and the Caribbean	$48,396,449 ($38,408,001–$59,672,173)	$120,717,047 ($95,789,339–$148,939,896)
India	$12,930,073 ($9,674,489–$16,499,072)	$63,422,693 ($47,576,673–$80,430,630)
World	$763,980,979 ($676,048,731–$857,982,275)	$1,918,318,955 ($1,700,574,632–$2,142,268,992)

*Income losses based on per capita gross national income (GNI) (Atlas method); CI, confidence interval. †Income losses based on purchasing power parity–adjusted per capita GNI.

### Livestock-associated Economic Losses

Estimated livestock-associated losses, with 95% CI, can be found in Table 6. Minimal annual losses, assuming liver condemnation alone with no correction for underreporting, is estimated at US $141,605,195 (95% CI, $101,011,553–$183,422,465). However, when losses from additional production factors (decreased carcass weight, decreased milk production, decreased hide value, decreased fecundity) are taken into account, losses range from US $1,249,866,660 (95% CI, $942,356,157 –$1,622,045,957), not taking into account underreporting, up to US $2,190,132,464 (95% CI, $1,572,373,055–$2,951,409,989), when underreporting is considered.

**Table 6:** Global annual cystic echinococcosis–associated livestock production losses

Category	Economic losses (95% CI) (US $)*
Liver condemnation†	$141,605,195 ($101,011,553–$183,422,465)
Decreased carcass weight†	$241,525,979 ($100,335,764–$518,035,773)
Decreased hide value‡	$34,871,148 ($23,965,776 – $46,162,828)
Decreased milk production§	$378,722,717 ($279,048,143–$495,682,356)
Decreased fecundity§	$453,141,617 ($278,287,046–$671,424,319)
Overall cost (no correction factor)	$1,249,866,660 ($942,356,157–$1,622,045,957)
Overall cost (adjusted for underreporting)	$2,190,132,464 ($1,572,373,055–$2,951,409,989)

*CI, confidence interval. †Sheep, goats, cattle, camels, pigs. ‡Sheep, cattle. §Sheep, goats, cattle.

## Discussion

**Figure 2:**
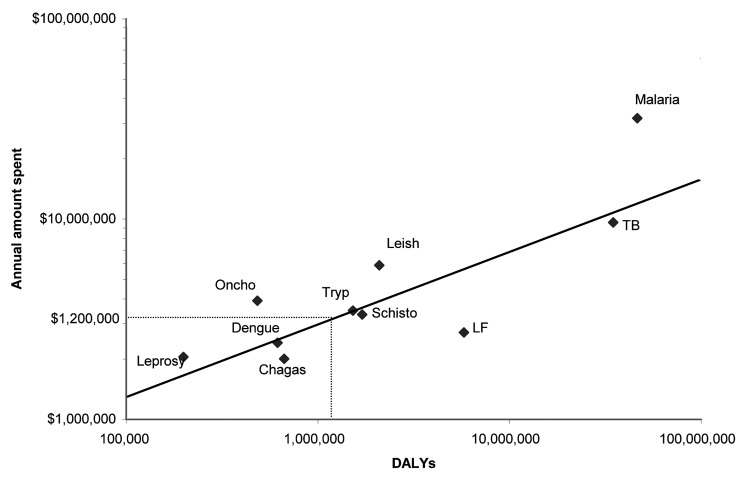
Annual budget (in US $) for diseases included in the United Nations Children's Fund/UNDP/World Bank/World Health Organization–supported Special Programme for Research and Training in Tropical Diseases (TDR) compared to their estimated global disability-associated life years (DALYs). The thinner lines indicate estimated DALYs lost because of cystic echinococcosis (CE) and the recommended funding level based on the TDR 2004–2005 approved program budget (Oncho, onchocerciasis; Tryp, trypanosomiasis; Schisto, schistosomiasis; Leish, leishmaniasis; LF, lymphatic filariasis; TB, tuberculosis). This figure does not take into account the substantial regional variability in both the estimates of DALYs lost and the annual budget for the diseases illustrated.

Even without correcting for the underreporting of human and livestock cases, CE has a substantial global disease impact in terms of DALYs and monetary losses. The importance of using both indicators is illustrated by the proportional difference in DALYS lost versus economic losses per region (Tables 4 and 5). If only monetary losses were evaluated, the severity of the situation in poorer regions would be underestimated because of the decreased income and economic value of livestock products relative to more economically prosperous regions. For example, China is responsible for 40% of the world's CE DALYs but only 19% of human-associated economic losses. However, losses based on ppp-adjusted per capita GNI give a better picture of the relative distribution of disease impact (Table 5). When the number of DALYs lost, taking into account the recognized underreporting of human cases, is compared with those of other parasitic conditions evaluated by the World Health Organization (WHO), worldwide losses due to CE are slightly less than those caused by African trypanosomiasis (1,525,000) and more than those caused by onchocerciasis (484,000) or Chagas disease (667,000) (*37*). Even though estimated number of DALYs lost from CE is greater than estimated losses from multiple members of the tropical disease cluster, CE continues to be excluded from funding associated with conditions related to low socioeconomic status. This exclusion best illustrated by evaluating research and training funding provided by the United Nations Children's Fund (UNICEF)/United Nations Development Programme (UNCP)/World Bank/WHO-supported Special Programme for Research and Training in Tropical Diseases (TDR). If funding for CE were placed on the same scale as TDR-supported diseases, based on estimated DALYs lost, CE should receive approximately US $1,200,000 annually (Figure 2) (*38*). For now, however, CE continues to be widely underappreciated by most international agencies. These findings emphasize the need for CE to be taken seriously as a global public health condition, regardless of its economic implications. What makes this disease exceptional, however, is that it is not only a substantial human health problem but also has a considerable economic effect on the livestock industries of some of the most socioeconomically fragile countries.

In addition to reporting the estimated global burden of CE, this study has shown the need for more accurate reporting of infected humans and livestock. Very few country-specific estimations of the true incidence of CE in humans have been made and no studies, to the authors' knowledge, that estimate its true prevalence in livestock (*16**,**17*). Presentation of the substantial economic losses for both the public health and agricultural sectors will also, we hope, encourage countries and international organizations to more closely examine potential control programs and cost-sharing methods between the 2 affected sectors (*10*).

The values presented in this paper are not definitive but instead estimates of the severity of the global situation from human- and livestock-associated CE. Considerable sums of money have been invested in the investigation and control of such parasitic conditions as lymphatic filariasis and onchocerciasis. Although these conditions can result in severe human disease, unlike CE they do not have severe secondary economic implications, such as massive livestock production losses (*39**,**40*). In addition, regional control programs that have been implemented and recommended thus far for CE, based on combinations of dog deworming, stray dog culling, sheep and goat vaccination, and education programs, have been shown to be very cost effective (*10**,**27*). CE is, therefore, a worthy condition for research and control program implementation, with substantial anticipated return on invested funding.
